# Through Ageing, and Beyond: Gut Microbiota and Inflammatory Status in Seniors and Centenarians

**DOI:** 10.1371/journal.pone.0010667

**Published:** 2010-05-17

**Authors:** Elena Biagi, Lotta Nylund, Marco Candela, Rita Ostan, Laura Bucci, Elisa Pini, Janne Nikkïla, Daniela Monti, Reetta Satokari, Claudio Franceschi, Patrizia Brigidi, Willem De Vos

**Affiliations:** 1 Department of Pharmaceutical Sciences, University of Bologna, Bologna, Italy; 2 Functional Foods Forum, University of Turku, Turku, Finland; 3 Division of Microbiology and Epidemiology, Department of Basic Veterinary Medicine, University of Helsinki, Helsinki, Finland; 4 Department of Experimental Pathology and CIG-Interdepartmental Center L. Galvani, University of Bologna, Bologna, Italy; 5 Department of Experimental Pathology and Oncology, University of Florence, Florence, Italy; 6 Laboratory of Microbiology, Wageningen University, Wageningen, The Netherlands; University of Hyderabad, India

## Abstract

**Background:**

Age-related physiological changes in the gastrointestinal tract, as well as modifications in lifestyle, nutritional behaviour, and functionality of the host immune system, inevitably affect the gut microbiota, resulting in a greater susceptibility to infections.

**Methodology/Principal Findings:**

By using the Human Intestinal Tract Chip (HITChip) and quantitative PCR of 16S rRNA genes of Bacteria and Archaea, we explored the age-related differences in the gut microbiota composition among young adults, elderly, and centenarians, i.e subjects who reached the extreme limits of the human lifespan, living for over 100 years. We observed that the microbial composition and diversity of the gut ecosystem of young adults and seventy-years old people is highly similar but differs significantly from that of the centenarians. After 100 years of symbiotic association with the human host, the microbiota is characterized by a rearrangement in the Firmicutes population and an enrichment in facultative anaerobes, notably pathobionts. The presence of such a compromised microbiota in the centenarians is associated with an increased inflammatory status, also known as inflammageing, as determined by a range of peripheral blood inflammatory markers. This may be explained by a remodelling of the centenarians' microbiota, with a marked decrease in *Faecalibacterium prauznitzii* and relatives, symbiotic species with reported anti-inflammatory properties. As signature bacteria of the long life we identified specifically *Eubacterium limosum* and relatives that were more than ten-fold increased in the centenarians.

**Conclusions/Significance:**

We provide evidence for the fact that the ageing process deeply affects the structure of the human gut microbiota, as well as its homeostasis with the host's immune system. Because of its crucial role in the host physiology and health status, age-related differences in the gut microbiota composition may be related to the progression of diseases and frailty in the elderly population.

## Introduction

Ageing is defined as “the regression of physiological function accompanied by the development of age” [1]. With a global impact on the physiology of the intestinal tract, the ageing process can seriously affect the composition of the human gut microbiota. The decreased intestinal motility results in a slower intestinal transit that affects defecation and leads to constipation [2]. The subsequent reduced bacterial excretion alters the gut fermentative processes in an unfavourable way [3], [4]. Inevitably, this affects the homeostasis of the bacterial ecosystem in the intestinal tract. Moreover, the age-related decline in the functionality of the immune system (immunosenescence) [5] is characterized by a chronic low-grade inflammatory status (inflammageing) [6]–[8]. In a healthy intestinal tract, the microbiota and the gut-associated immune system are assumed to share a fine and dynamic homeostatic equilibrium [9]. The inflammageing process can undermine this balance, leading to changes in intestinal microbial structure and composition [10]. Finally, considering the impact of the diet on the gut microbiota composition [11], changes in nutritional behaviour and life style of the aged people concur to the age-related unbalances of the intestinal microbial community.

Coevolved with the human host and its ancestors for millions of years, the intestinal microbiota contributes significantly to our physiology, among others by supplying nutrients and protecting against pathogens [9]. This has led to the hypothesis that age-related changes in the composition of the symbiotic microbiota may contribute to the progression of diseases and frailty in the elderly [10], [12]. The reported age-related differences in the intestinal microbiota composition include increase in the total number of facultative anaerobes and shifts in the dominant species within several bacterial groups, whereas no significant changes are reported in the total number of anaerobic bacteria [13]–[18]. The majority of earlier studies has relied on cultivation-based approaches but high-throughput culture-independent molecular techniques are considered essential to provide a global insight in the intestinal microbiota [19]. A 16S rRNA gene-based diversity microarray for the characterization of the human gut microbiota (Human Intestinal Tract Chip, HITChip) has been recently developed and validated [20]. The HITChip was found to be comparable but faster than deep pyrosequencing [21], indicating that it is among the most powerful tools presently available for characterizing the human gut microbiota. A pilot study with five adults and five elderly subjects using HITChip analysis showed the structure and composition of the gut microbiota to be different in different age groups [20], [21].

In this study, we undertook to explore the age-related differences both in the inflammatory status and in the gut ecosystem composition, by using HITChip analysis. In particular, we decided to expand the usual target populations of comparative studies, addressing not only young adults (20–40 years old) and elderly (60–80 years old), but also centenarians, representative of a group of people who reached the extreme limits of the human lifespan [22]. This approach aims at the broadening of our view of the differences in the gut microbiota of adult human beings of increasing age.

## Results

### Fecal microbiota profiles of centenarians, elderly and young adults

**Figure 1:**
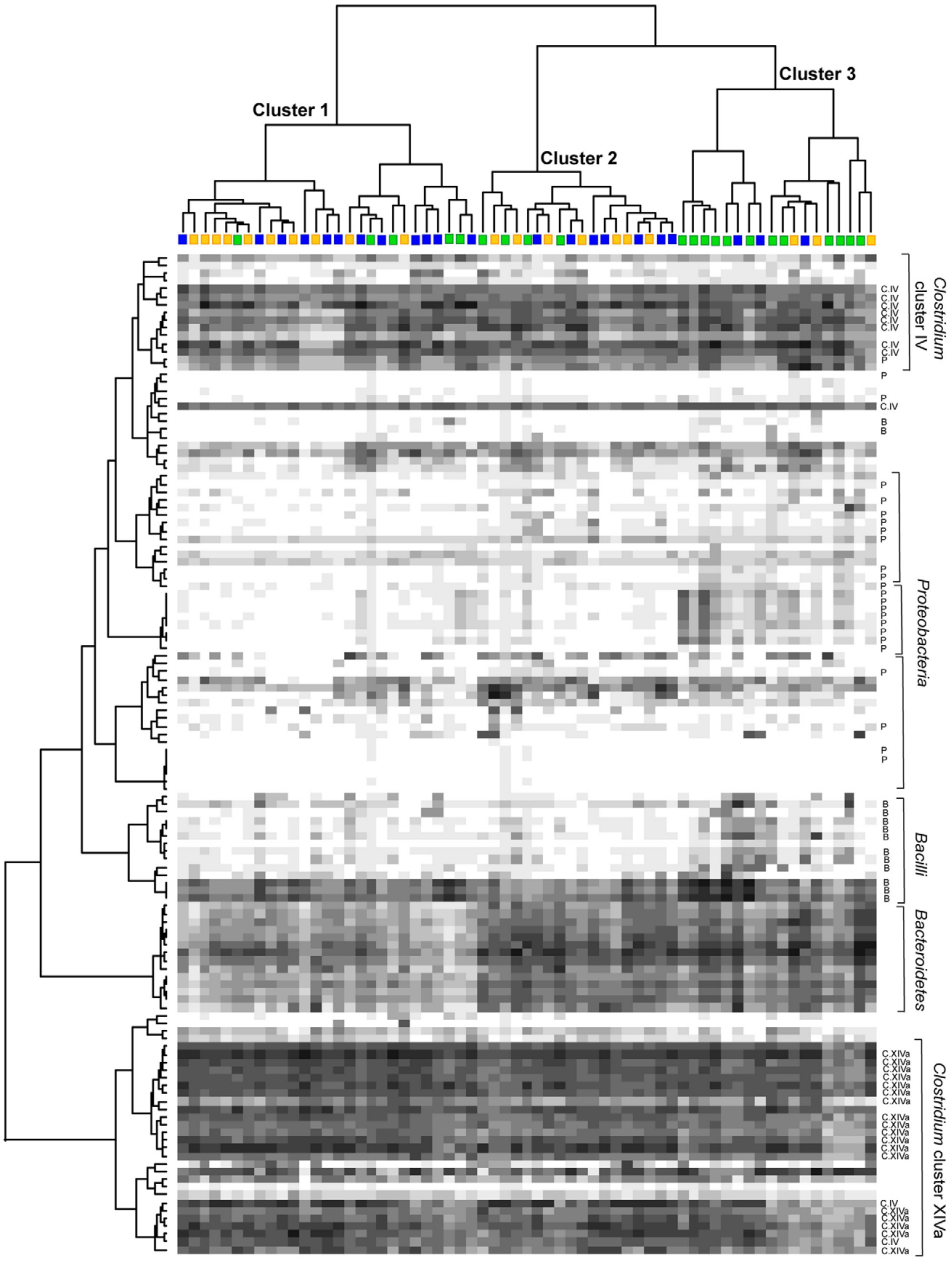
Hierarchical clustering, with heat map, of the HITChip profiles of centenarians, elderly and young adults. Subjects belonging to the groups C, E, and Y are indicated by green, blue, and yellow squares, respectively. Darkness of the spot corresponds to the bacterial abundance in the sample. Pearson correlation and Ward's clustering method were used. Genus-like bacterial groups belonging to Proteobacteria (P), Bacilli (B), *Clostridium* cluster IV (C.IV) and XIVa (C.XIVa) are indicated. The two phylogenetic groups members of the *Clostridium* cluster IV located within the *Clostridium* cluster XIVa (bottom) are *Faecalibacterium prausnitzii et rel.* and *Papillibacter cinnamovorans et rel*.

The fecal microbiota of centenarians (group C), elderly (E) and young adults (Y) was characterized using the HITChip [20]. Hybridization was performed with at least 2 replicates for each sample, and reproducibility was tested by calculating Pearson's correlation coefficient (Pearson's *r*) and Relative Standard Deviation (RSD). For each subject a phylogenetic fingerprint of the intestinal microbiota was obtained. Pearson's *r* ranged between 0.982 and 0.999, with a mean value of 0.993, showing that the HITChip profiles were highly reproducible, and the RSD ranged between 1.8% and 13.2%, with a mean value of 5.1%. Hierarchical clustering of the genus-like taxa in a heat map showed that centenarians tended to group together, whereas no separation was observed with the subjects belonging to groups E and Y (Fig. 1). The proportion of centenarians in Cluster 3 (67%) was found to be significantly higher than in the other clusters (χ^2^ test, P value = 0.001). Clusters 1 and 2 contain similar proportions of each age groups (18% C, 44% E, 39% Y, for Cluster 1; 22% C, 38% E, 38% Y, for Cluster 2). The same results were obtained by using analysis of similarity (ANOSIM, [23]), which showed that the microbiota profiles of centenarians are significantly different (P = 0.001, R value = 0.32) from that of all younger people (E and Y). The gut microbiota of subjects in Cluster 3 is characterized by higher amounts of Proteobacteria and Bacilli, and decreased amounts of *Clostridium* cluster XIVa bacteria. Finally, Bacteroidetes showed lower intensities in cluster 1, which is composed by mixed subjects, highlighting that the decrease in Bacteroidetes amount in the gut microbiota does not seem to be related to the ageing.

**Figure 2:**
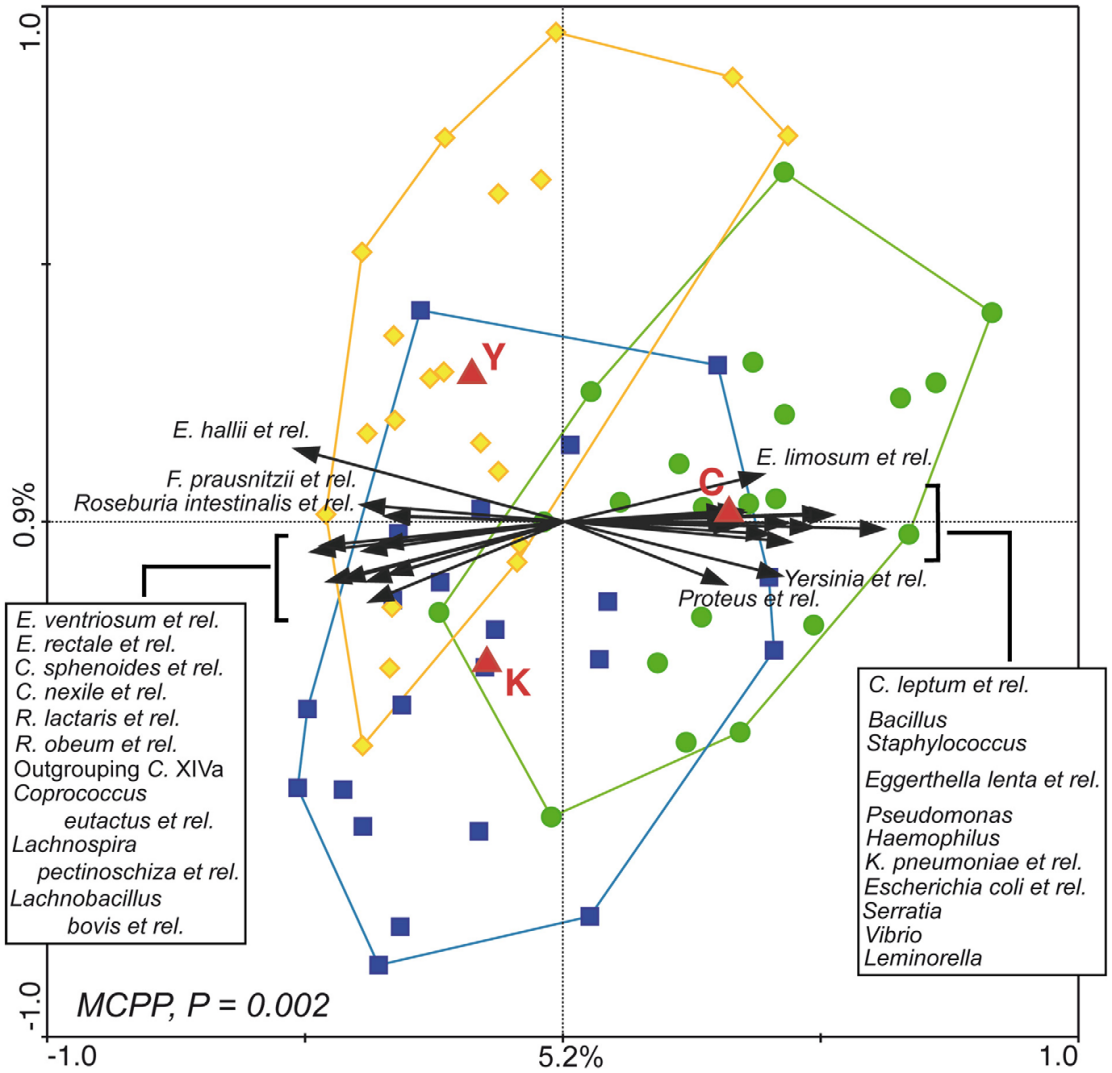
Triplot of the RDA of the microbiota composition of centenarians, elderly and young adults. Subjects belonging to group C, E, and Y are indicated by green circles, blue squares and yellow diamonds, respectively. Constrained explanatory variables (C, E, and Y) are indicated by red triangles. Responding bacterial subgroups that explained more than 10% of the variability of the samples are indicated by black arrows. First and second ordination axes are plotted, showing 5.2% and 0.9% of the variability in the dataset, respectively. Log transformed data were used for the analysis. Bottom-left, P value obtained by MCPP is reported. Abbreviations: C., *Clostridium*; E., *Eubacterium*; F., *Faecalibacterium*; R., *Ruminococcus*; K., *Klebsiella*.

In order to highlight the bacterial groups principally responsible for the age-related differences in the gut microbiota structure, a multivariate analysis was carried out using the age groups C, E, and Y as nominal “environmental variables”, and the log-transformed average hybridization signal for the 129 genus-like phylogenetic groups as “species variables”. The resulting ordination plot shows the redundancy analysis (RDA), which focuses on the major carriers of the difference among the groups of subjects (Fig. 2). The differences shown in RDA are significant (P = 0.002) as assessed by Monte Carlo Permutation Procedure (MCPP). A total of 6.1% of the total variation in the dataset (microbiota composition at genus-like level) could be related to the environmental variables (age-groups). Most of this variation (5.2%) is plotted on the first axis that separates the centroid of group C from the other centroids (E and Y), plotted on a line along the second axis, which shows only 0.9% of variability. This analysis indicated that the differences in the microbiota fingerprint between centenarians and all the other subjects are greater than the differences between elderly and young adults. The samples belonging to groups C and Y form almost separate clusters in RDA, whereas the group E cluster overlaps with both of them. Confirming the heatmap results, RDA showed that the fecal microbiota of centenarians is enriched in many facultative anaerobes, mostly belonging to Proteobacteria (*Escherichia coli et rel., Haemophilus, Klebsiella pneumoniae et rel., Leminorella, Proteus et rel., Pseudomonas, Serratia, Vibrio*, and *Yersinia et rel*.), and Bacilli (*Bacillus, Staphylococcus*). On the other side of the plot, higher amounts of many bacterial groups belonging to *Clostridium* cluster XIVa seemed to characterize the fecal microbiota of E and Y samples. Remarkably, the arrows corresponding to the phylogroups *Faecalibacterium prausnitzii et rel*. and *Clostridium leptum et rel.*, both belonging to the *Clostridium* cluster IV, pointed in opposite directions, indicating that *F. prausnitzii* group decreased in centenarians whereas *C. leptum* group increased.

Since 95% of subjects in group C were females, the RDA described above was repeated excluding male subjects from groups E and Y. This restriction did not change the overall shape of the plot (data not shown), suggesting that gender differences play no or marginal role in the effect of ageing on the gut microbiota composition.

Subsequently, the diversity of the microbial communities in the different age groups was assessed. The diversity discovered by the HITChip was expressed as Simpson reciprocal index of diversity. Simpson indices obtained for group C (127.0±54.2) were significantly lower than those obtained for E (149.4±40.5, P = 0.02) and Y (162.8±35.1, P = 0.002), indicating that the microbiota of centenarians is significantly less diverse than that of elderly or young adults. On the contrary, the difference between Simpson indices obtained for E and Y was not statistically significant.

### Quantitative differences between the gut microbiota of centenarians, elderly and young adults

**Figure 3:**
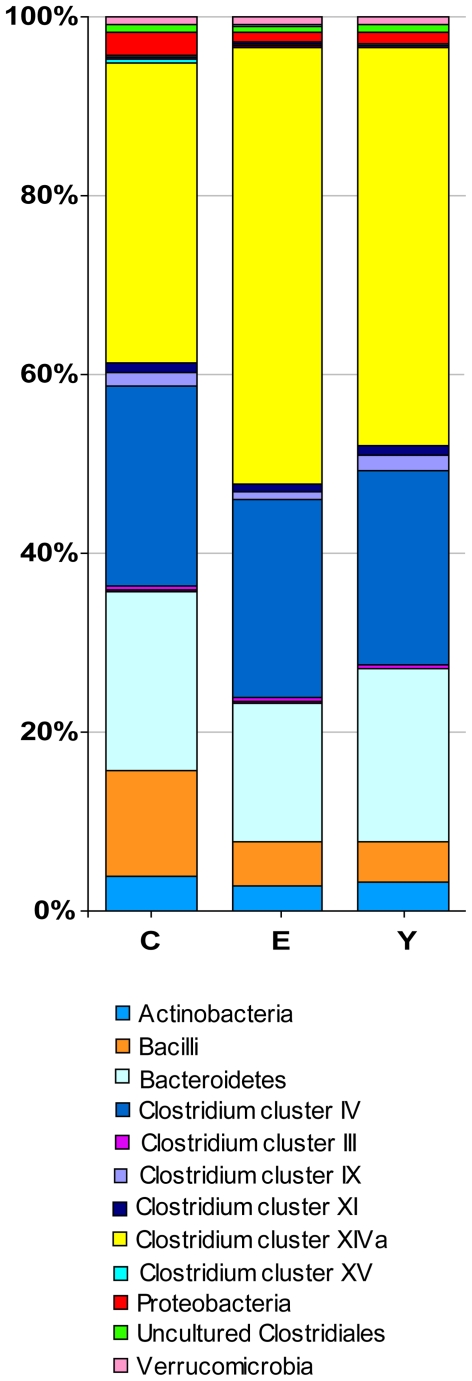
Relative contribution of the phylum/order-like phylogroups to the microbiota of centenarians, elderly and young adults. In the legend, phylum/order-like phylogroups which contribute for at least 0.5% to one of the profiles are indicated.

The relative contributions of the major phyla were assessed by analysing the phylum/order taxa in the fecal microbiota of the subjects in groups C, E and Y (Fig. 3). Together, the Bacteroidetes and Firmicutes contributed to the fecal microbiota for the 93% in the case of centenarians, and 95% in both groups E and Y. Bacteroidetes contributed for 20, 16, and 19% to the total microbiota of subjects belonging to groups C, E and Y, respectively. The Firmicutes/Bacteroidetes ratios obtained for groups C, E and Y were 3.6, 5.1, and 3.9, respectively. To evaluate the significance of this difference, the sum of the hybridization signals of the probes which referred to the Bacteroidetes and the Firmicutes was calculated for each subject. The differences among groups of samples, in both the Bacteroidetes proportions and the Firmicutes/Bacteroidetes ratios, were not statistically significant (P = 0.73, and P = 0.55, respectively).

*Clostridium* cluster IV contributed for the 22% to the microbiota of all age groups. qPCR for *C. leptum* group, which is comparable to *Clostridium* cluster IV, showed no significant difference between centenarians and other age groups (Table 1), confirming the HITChip results. Conversely, qPCR showed group E to have significantly more bacteria belonging to *C. leptum* group when compared to Y (P = 0.05), although no statistical difference in HITChip signals was observed. The relative contribution of *Clostridium* cluster XIVa is clearly lower in centenarians (34%) than in the other groups (49%, group E; 44%, group Y). By comparing the sum of the hybridization signals of the probes which referred to *Clostridium* cluster XIVa in the C and E groups, and in the C and Y groups, P values of 0.001 and 0.02 were obtained, respectively. The proportion of Bacilli is significantly higher in centenarians (12%), if compared to groups E (5%, P = 0.05) and Y (5%, P = 0.03). Centenarians also tended to have a higher proportion of Proteobacteria (2.6%) with respect to E (1.2%, P = 0.06) or Y (1.2%, P = 0.07).

**Table 1:** Quantitative PCR results for bacterial groups.

	Average 16S rRNA gene copies/ug fecal DNA			Ratio^A^			P values^B^		
Bacterial species or group	C	E	Y	C/E	C/Y	E/Y	C *vs* E	C *vs* Y	E *vs* Y
*Clostridium leptum* group	1.99E+07	3.02E+07	1.60E+07	0.7	1.3	1.9	0.3	0.4	**0.05**
*Faecalibacterium prausnitzii*	2.01E+07	6.99E+07	3.80E+07	0.3	0.5	1.8	**0.02**	0.5	0.1
*Bifidobacterium* spp.	6.57E+08	6.87E+08	9.50E+08	1.0	0.7	0.7	0.7	**0.02**	0.1
*Akkermansia*	2.75E+06	1.45E+06	1.22E+06	1.9	2.2	1.2	0.3	0.2	**0.01**
Archaea	1.98E+06	1.48E+06	4.58E+05	1.3	4.3	3.2	0.2	0.2	0.8
Universal (total bacteria)	1.78E+09	1.39E+09	1.38E+09	1.3	1.3	1.0	0.4	0.4	1

A Ratios of bacterial 16S rRNA gene counts between groups C, E and Y.

B In bold, the most significant changes (P values<0.05) are reported.

To provide a more detailed view of the age-related differences in the core gut microbiota composition, quantitative analysis was focused on the genus-like bacterial groups having 100% of prevalence (defined as percentage of positive samples in each study group) in all the age groups. This core microbiota was constituted principally of Bacteroidetes and Firmicutes, mostly *Clostridium* clusters XIVa and IV. Several genus-like groups showed a significantly different abundance in groups C, E and Y, while others showed a tendency (P = 0.05–0.08) of increased or decreased abundance in the different age groups (Table 2). In accordance with the RDA result (Fig. 2), the vast majority of the significant differences in bacterial groups were found when group C was compared to E and Y. Relative contribution of each genus-like bacterial group to the total microbiota was calculated for the three groups of subjects. The ratio of the average hybridization signals obtained for groups C and E, and C and Y, was determined to better highlight the increase or decrease of each genus-like group at the different stages of life. Most of the bacteria that significantly decreased in group C with respect to both E and Y belonged to *Clostridium* cluster XIVa. In *Clostridium* cluster IV, *Papillibacter cinnamovorans et rel*., and *F. prausnitzii et rel.* showed a significant decrease in centenarians, while *C. leptum et rel., Sporobacter termiditis et rel., Anaerotruncus colihominis et rel., Clostridium orbiscindens et rel.* showed a significant increase. The decrease of *F. prausnitzii* in centenarians with respect to the other two age groups was confirmed by qPCR analysis (Table 1). Considering that the relative proportion of *Clostridium* cluster IV in the total microbiota remained unaffected by the age of the subjects (Fig. 3, Table 1), the analysis at genus-like level suggests a rearrangement in the composition of this bacterial cluster, in terms of genera or species, in centenarians. In line with the RDA results (Fig. 2), the average amounts of all the bacteria listed above did not significantly differ between E and Y groups. Differences between E and Y were found only for *Acquabacterium* (P = 0.05), *Collinsella et rel.* (P = 0.04), and *Dialister et rel*. (P = 0.02). These phylogenetic groups, belonging to Proteobacteria, Actinobacteria and *Clostridium* cluster IX, respectively, are not highly represented in the human gut. Moreover, for all the bacterial groups showing significant differences between centenarians and all the other subjects, the HITChip signal ratios C/E and C/Y were often very similar (Table 2), suggesting that the changes in the composition of the gut microbiota are not linearly correlated with the age.

**Table 2:** Genus-like bacterial groups that were found to differ significantly between centenarians (C), elderly (E) and young adults (Y).

		Relative contribution (%)^A^			Ratio^B^		P value^C^	
Phylum/order	Genus-like phylogenetic group	C	E	Y	C/E	C/Y	C *vs* E	C *vs* Y
*Clostridium* cluster XV	*Eubacterium limosum et rel.*	0.35	0.02	0.02	16	14.5	0.0009	0.01
*Proteobacteria*	*Klebsiella pneumoniae et rel.*	0.17	0.03	0.02	5.3	6.7	0.002	0.0009
	*Vibrio*	0.15	0.03	0.03	5.4	5.4	<0.0001	<0.0001
	*Enterobacter aerogenes et rel.*	0.05	0.03	0.02	1.9	2.1	0.03	0.04
*Actinobacteria*	*Eggerthella lenta et rel.*	0.11	0.06	0.04	1.8	2.7	0.02	0.0001
*Bacilli*	*Bacillus*	0.07	0.05	0.03	1.4	2.0	0.01	0.04
*Clostridium* cluster IV	*Clostridium leptum et rel.*	2.37	1.27	1.33	1.8	1.8	0.006	0.005
	*Sporobacter termiditis et rel.*	1.14	0.75	0.70	1.5	1.6	0.05	0.04
	*Anaerotruncus colihominis et rel.*	0.99	0.68	0.66	1.4	1.5	*0.08*	0.01
	*Clostridium orbiscindens et rel.*	1.52	1.05	1.16	1.4	1.3	0.03	*0.08*
	*Faecalibacterium prausnitzii et rel.*	2.01	4.05	4.24	0.5	0.5	0.01	0.006
	*Papillibacter cinnamovorans et rel.*	1.30	1.72	1.80	0.7	0.7	*0.06*	0.04
*Clostridium* cluster XIVa	*Clostridium colinim et rel.*	0.90	1.98	1.57	0.4	0.6	*0.06*	0.05
	*Clostridium sphenoides et rel.*	0.97	1.92	1.50	0.5	0.6	0.0002	0.003
	*Eubacterium hallii et rel.*	3.16	4.75	5.76	0.7	0.5	0.03	0.004
	*Eubacterium rectale et rel.*	1.68	3.61	3.02	0.5	0.5	0.001	0.004
	*Eubacterium ventriosum et rel*	1.21	2.77	2.62	0.4	0.4	0.0005	0.0002
	*Lachnobacillus bovis et rel.*	1.15	1.98	1.46	0.6	0.8	0.007	0.03
	Outgrouping *Clostridium* cluster XIVa	0.63	0.94	1.04	0.7	0.6	0.02	0.01
	*Roseburia intestinalis et rel.*	1.57	3.04	3.21	0.5	0.5	0.006	0.03
	*Ruminococcus lactaris et rel.*	0.65	1.07	0.87	0.6	0.7	0.002	0.01
	*Ruminococcus obeum et rel.*	1.73	2.79	2.65	0.6	0.6	0.003	0.01

A Relative contributions of genus-like phylogenetic group to the fecal microbiota was calculated as percentage of signal intensities the total signal intensity.

B Ratio of the average relative abundance of each genus-like phylogenetic group calculated for subjects belonging to C and E groups, and to C and Y groups. Bacterial groups showing C/E and C/Y ratio <1 decreased in the subjects of group C.

C In italic, relevant groups with P values ranging from 0.05 to 0.08 (italics) are reported.

In addition to the qPCR analyses of *Clostridium* cluster IV and *F. prausnitzii*, performed to confirm the HITChip results, we quantified bifidobacteria and *Akkermansia muciniphila*, which in previous studies had shown to decrease in the old age (Table 1). The qPCR revealed that the amount of total bifidobacteria was significantly lower in C when compared to Y (P = 0.023). However, no statistical difference was detected in the *Bifidobacterium* signals in the HITChip. This discrepancy is likely due to the lower quantitative sensitivity of the HITChip, with respect to the qPCR, which detected a difference of only 0.3 log unit between groups C and Y. The amounts of *Akkermansia* spp. were comparable between centenarians and other age groups, both in qPCR and HITChip analysis. However, by qPCR, subjects in group Y were observed to harbour significantly less *Akkermansia* than group E, both in means of prevalence and amount of 16S rRNA gene copies. We also quantified by qPCR the Archaea, which are not targeted by the HITChip. The amount of archaeal DNA did not show any significant difference between centenarians and the other age groups (Table 1). Interestingly, centenarians tended to harbour Archaea more frequently than the elderly or young adults (prevalence 65%, 36% and 45%, respectively), although the difference was not significant.

### Microbiota comparison between centenarians and their offspring

In order to assess whether the microbiota of centenarians can be related to that of their offspring, the HITChip analysis was performed on samples from group F, composed of 21 offspring subjects of centenarians. The average age in group F was 67.5 years. The HITChip profile of each centenarian was compared to that of his/her offspring, and to the profile of randomly selected subjects belonging to the group E, and Pearson's *r* were calculated. A mean Pearson's *r* of 0.42±0.18 for the C-F pairs and 0.44±0.20 for the random C-E pairs were obtained. These results demonstrate that the gut microbiota composition of parents and offspring shows no significant similarity at the old age (P = 0.79).

The average Simpson indices obtained for group F was 151.7±49.3. The microbiota of elderly belongings to groups E and F did not differ in diversity as assessed by Simpson index (P = 0.87). Also, the proportions of the phylum/order-like phylogenetic groups in E and F subjects were comparable (data not shown). Furthermore, RDA analysis showed that samples of the F and E groups were mixed and the centroids were close to each other, whereas the centroid of group C was plotted distant from both of them (Supporting Fig. S1). No significant differences in the overall microbiota composition were found between groups F and E (MCPP, P = 0.39). The statistically significant differences in genus-like bacterial groups between C and E (Table 2) were also found between C and F (data not shown). In general, the microbiota of elderly people in group F seemed more similar to the microbiota of subjects in the age-matched group E, than to that of their centenarian parents. This indicates that centenarians have a unique microbial composition.

Phylotypes belonging to the major bacterial phyla of the human gut microbiota, such as *Clostridium* cluster IV and XIVa and Bacteroidetes, were present in all study subjects. On the other hand, some bacterial groups which are less represented in the human gut ecosystem, such as Fusobacteria and Proteobacteria, showed higher prevalence (the percentage of positive samples in each study group) in centenarians and their offspring than in E or Y groups (Table 3). The most noticeable differences in the prevalence concerned *Campylobacter*, *Helicobacter* and *Fusobacterium*. This trend is mainly limited to opportunistic or potentially pathogenic bacterial groups, and it is likely to reflect the microbial exposure in the shared living environment and/or the close contacts between family members, since most of the subjects in group F (13 out of 21, defined as group FC) lived with their centenarian parents. Several of the phylogroups listed in Table 3, *Asteroplasma et rel., Peptostreptococcus micros et rel., Fusobacterium, Alcaligenes faecalis et rel., Campylobacter, Desulfovibrio et rel., Helicobacter, Leminorella, Moraxellaceae, Proteus et rel., Brachyspira*, showed higher prevalence values in group FC than in group FN, a subset of group F including the offspring subjects who did not live with their parents. This supports the explanation that the observed common microbiota between family members is due to the sharing of a common household.

**Table 3:** Genus-like bacterial groups which showed differences in terms of prevalence between groups C (centenarians), F (elderly offspring of the centenarians), E (elderly), and Y (young adults).

		Prevalence (%)				
Phylum/order	Genus-like phylogenetic group	C	F	(FC, FN)^A^	E	Y
*Actinobacteria*	*Corynebacterium*	90	90	(83, 100)	50	40
	*Micrococcaceae*	90	76	(83, 67)	59	35
*Asteroplasma*	*Asteroplasma et rel.*	48	38	(42, 33)	32	20
*Bacilli*	*Staphylococcus*	100	100	(100, 100)	91	85
*Clostridium* cluster XI	*Peptostreptococcus anaerobius et rel.*	76	100	(100, 100)	59	55
	*Peptostreptococcus micros et rel.*	52	52	(67, 33)	18	15
*Fusobacteria*	*Fusobacterium*	28	43	(50, 33)	5	5
*Proteobacteria*	*Alcaligenes faecalis et rel.*	71	81	(92, 67)	50	35
	*Bilophila et rel.*	90	86	(83, 89)	68	70
	*Campylobacter*	29	33	(50, 11)	5	5
	*Desulfovibrio et rel.*	90	90	(100, 78)	77	50
	*Escherichia coli et rel.*	95	81	(75, 89)	77	70
	*Haemophilus*	90	76	(75, 79)	68	55
	*Helicobacter*	33	33	(50, 11)	5	5
	*Leminorella*	95	76	(83, 67)	64	65
	*Moraxellaceae*	48	38	(58, 11)	18	10
	*Oceanospirillum*	57	76	(75, 78)	36	30
	*Proteus et rel.*	90	76	(83, 67)	41	35
	*Pseudomonas*	90	90	(83, 100)	77	45
	*Serratia*	100	95	(92, 100)	77	70
	*Yersinia et rel.*	95	86	(83, 89)	72	60
*Spirochaetes*	*Brachyspira*	19	38	(50, 22)	9	0

A Prevalence values of subsets of group F, FC (offspring who lived with their centenarian parents) and FN (offspring who do not live with their centenarian parents) are presented in between brackets.

### Immunophenotyping analysis

In order to assess the immune system status of the subjects, we performed a cytofluorimetric analysis of the major lymphocytes subsets, with particular attention to the T cell compartment. No significant differences between the groups E and F were observed regarding both T and B cell subpopulations (data not shown). Hence we grouped all these elderly subjects (designated group S, for Seventy years which is the average age of these subjects) and compared their immune status with that of the other groups (Table 4). A significant, progressive age related decrease of naïve T cells was observed with a concomitant significant increase of memory T cells, as was expected as characteristic features of immunosenescence.

**Table 4:** Immunophenotypical analysis of the main lymphocyte subsets involved in immunosenescence.

		Mean (%)± S.E.M^A^			P value^B^		
Lymphocyte subsets		C	S	Y	C *vs* S	S *vs* Y	C *vs* Y
Naïve T lymphocytes (CD45RA+CCR7+)	T helper	15.3±2.5	22.5±1.3	33.1±2.0	0.008	<0.0001	<0.0001
	T cytotoxic	12.9±1.4	15.7±1.2	33.9±2.3		<0.0001	<0.0001
Central Memory T lymphocytes (CD45RA-CCR7+)	T helper	34.8±3.5	21.5±1.9	13.7±1.1	<0.001	0.009	<0.0001
	T cytotoxic	11.4±1.2	9.8±1.5	5.0±0.6		0.03	<0.0001
Effector Memory T lymphocytes (CD45RA-CCR7-)	T helper	42.2±2.8	40.8±1.7	37.8±2.2			
	T cytotoxic	47.3±3.5	42.5±2.0	33.4±2.3		0.007	0.002
Terminal Effector T lymphocytes (CD45RA+CCR7-)	T helper	7.6±1.1	15.2±1.6	15.4±0.7	0.03		<0.0001
	T cytotoxic	28.5±3.9	32.0±2.6	27.7±2.2			
Effector T lymphocytes (CD28−)	T helper	10.7±2.0	9.5±1.6	3.5±1.2		<0.001	0.001
	T cytotoxic	48.2±5.4	49.8±3.7	28.6±4.4		0.002	0.02
Activated T lymphocytes (CD25+)	T helper	26.6±2.6	23.8±1.5	29.2±1.8		0.03	
	T cytotoxic	13.3±2.9	6.6±0.7	5.5±0.4	0.003		0.001

A Results are expressed as mean percentage of T helper and T cytotoxic lymphocytes ± S.E.M (standard error of the mean).

B P values <0.05 are reported.

### Evaluation of the inflammatory status

To explore the inflammation level of the subjects involved in this study, plasma levels of the major pro- and anti-inflammatory cytokines were evaluated. The pro-inflammatory cytokines IL-6 and IL-8 increased in C with respect to S and Y. In the case of IL-6 the increase was significant (C*vs*S, P = 0.003; C*vs*Y, P = 0.05). On the contrary, IL-1α and TNF-α levels were significantly lower in C in comparison to Y (IL-1α, P = 0.03; TNF-α, P = 0.03). IL-1β and IFN-γ levels did not change significantly in the different age groups. Regarding anti-inflammatory cytokines (TGF-β1 and IL-10), no significant differences were observed among the age groups. Moreover, plasma levels of IL-2 and IL-12 which play an important role in activating the immune response, showed a decrease in C with respect to S and Y, which was significant in the case of IL-2 (C*vs*Y, P = 0.04).

The calculation of the Inflammation Score (IS) highlighted that most of the centenarians (66.7%) were characterized by highly inflamed status, whereas in groups S and Y the majority of the subjects fell in the “low inflammation” group (S, 69.2%; Y, 75%). The difference in the proportions of highly inflamed subjects was significant when groups C and S (χ^2^ test, P = 0.02), and C and Y (χ^2^ test, P = 0.02) were compared. Average IS values of 4.8, 4.0, and 3.3 were obtained for groups C, S, and Y, respectively.

### Correlation between cytokine levels and gut microbiota

**Figure 4:**
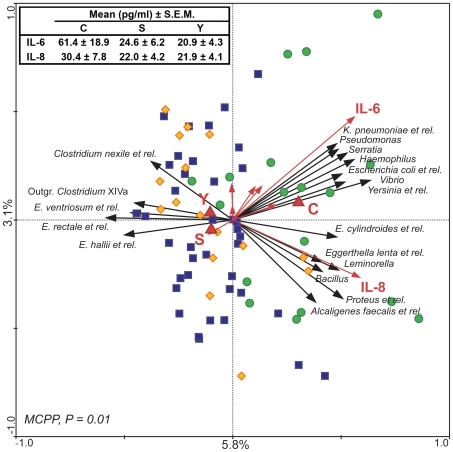
Correlation between microbiota composition and plasma levels of pro-inflammatory cytokines. In the RDA blood cytokine levels (red arrows) and age groups (C, S, and Y, red triangles) are used as linear and nominal environmental variables, respectively. Samples belonging to C, S and Y groups are indicated by green circles, blue squares and yellow diamonds, respectively. Responding bacterial subgroups that explained more than 20% of the variability of the samples are indicated by black arrows. First and second ordination axes are plotted, showing 5.8% and 3.1% of the variability in the dataset, respectively. Red arrows which are not labelled corresponds to (clockwise, starting from the left) TNF-α, IFN-γ, IL-2, IL-1α, IL-12p70, and IL-1β. Log transformed data were used for this analysis. Bottom-left, P value obtained by MCPP is reported. Top-left, average blood levels of IL-6 and IL-8 in groups C, S and Y are reported.

To find possible correlations between the microbiota composition and the cytokines pattern, log-transformed results of pro-inflammatory cytokines quantification and HITChip profiling of the gut microbiota were used in a multivariate analysis, using cytokines plasma levels and the age groups as “environmental variables”. RDA shows that 8.9% of the total variability of the gut microbiota can be related to the pro-inflammatory cytokines pattern (Fig. 4). Relations shown in the plot are statistically significant, as established by MCPP (P = 0.014). In accordance with previous analysis (Fig. 2), the centroid of group C is plotted distant from both the S and Y centroids, highlighting the similarity in the gut microbiota asset and the relation with the inflammatory status between elderly and young adults. Several bacteria belonging to the phylum Proteobacteria seemed to be positively correlated with IL-6 and IL-8. IL-8 was correlated with *Alcaligenes faecalis et rel.*, *Leminorella*, and *Proteus et rel.*, while IL-6 was correlated with *Escherichia coli et rel., Haemophilus, Klebsiella pneumoniae et rel., Pseudomonas, Serratia, Yersinia et rel.*, and *Vibrio*. IL-8 and IL-6 were correlated also with *Bacillus* (Bacilli), *Egghertella lenta et rel.* (Actinobacteria), and *Eubacterium cylindroides et rel. (Clostridium* cluster XIVa). On the other side, *Eubacterium hallii et rel., Eubacterium ventriosum et rel., Eubacterium rectale et rel.*, *Clostridium nexile et rel.*, and Outgrouping *Clostridium* cluster XIVa (all belonging to the *Clostridium* cluster XIVa) are inversely correlated with IL-6 and IL-8.

Pearson's correlation analysis of the HITChip data and cytokines revealed significant Pearson's *r* for IL-6 and IL-8 (Table 5). All the bacterial groups which exhibited a slight positive correlation with either IL-6 or IL-8 (ranging between 0.41 and 0.55) belonged to the phylum Proteobacteria. Interestingly, only one bacterial group, *Ruminococcus lactaris et rel.* (*Clostridium* cluster XIVa), has been found with a slight negative correlation with IL-8 (−0.44, P = 0.0001).

**Table 5:** Relevant positive and negative Pearson's correlations of specific genus-like bacterial groups and the pro-inflammatory cytokines IL-6 and IL-8.

	IL6		IL8	
	Pearson's *r*	P value	Pearson's *r*	P value
*Alcaligenes faecalis et rel.*	/	/	0.43	<0.001
*Escherichia coli et rel.*	0.46	<0.0001	0.35	0.003
*Haemophilus*	0.47	<0.0001	0.24	0.04
*Klebsiella pneumoniae et rel.*	0.47	<0.0001	/	/
*Leminorella*	0.26	0.02	0.41	<0.0001
*Proteus*	0.32	0.007	0.55	<0.0001
*Pseudomonas*	0.45	<0.0001	/	/
*Serratia*	0.45	<0.0001	0.23	0.05
*Vibrio*	0.45	<0.0001	0.28	0.02
*Yersinia*	0.48	<0.0001	0.33	0.005
*Ruminococcus lactaris et rel.*	−0.32	0.006	−0.44	<0.001

## Discussion

In this paper we addressed the age-related differences in both the gut microbiota and the inflammatory status among different stages of the whole adult life, starting from young adults (30 years old in average), through elderly (70 years old), to the extreme limit of the human lifespan, represented by a group of centenarians (100 years old). The enrolled subjects were phenotypically well characterized for their physical and cognitive health status. They represent the general population of a restricted geographic area of Northern Italy and can be considered as a relatively homogeneous cohort regarding lifestyle and dietary habits.

The uniqueness of this comparative study resides in the introduction of this third, extremely aged, group, alongside to the young and elderly adults that have been addressed by earlier studies [14], [15], [17], [20]. The analysis of the microbiota that had been coexisting with their host for over 100 years allowed us to highlight that changes in the gut ecosystem, in terms of composition and diversity, do not follow a linear relation with age. In fact, the difference between the gut microbiota of young adults and elderly, separated by more than 40 years in average, is remarkably small when compared to that observed between centenarians and elderly, separated by less than 30 years of life.

Young and elderly adults showed a very comparable overall structure of the gut microbiota, which confirms the most recent characterizations, with Bacteroidetes and Firmicutes highly dominant (contributing for approximately 95% to the microbiota), and smaller fractions of Actinobacteria, and Proteobacteria [24]–[26]. The diversity values of the gut microbiota of elderly and young adults were found comparable (average Simpson index 149.3 and 162.8, respectively), and fits the range of diversity expected for healthy adults (Simpson index  = 150–200) [27].

In contrast, centenarians stand out as a separate population, whose microbiota shows an organization which significantly differs from the adult-like pattern, and a low diversity in terms of species composition (Simpson index  = 127). Bacteroidetes and Firmicutes still dominate the gut microbiota of extremely old people (representing over 93% of the total bacteria). However, in comparison to the younger adults, specific changes in the relative proportion of Firmicutes subgroups were observed, with a decrease in the contributing *Clostridium* cluster XIVa, an increase in Bacilli, and a rearrangement of the *Clostridium* cluster IV composition. Moreover, the gut microbiota of centenarians is enriched in Proteobacteria, a group containing many of those bacteria recently redefined as “pathobionts” [28], [29]. These are considered to be minor and opportunistic components of the human gut ecosystem that, under some circumstances, e.g. inflammation, may escape surveillance, overtake mutualistic symbionts and induce pathology.

The structure of the gut microbiota is well known to be relatively stable through adulthood [30], [31]. Our findings suggest that this stability may last longer than expected, and that the ageing process starts to affect the gut microbiota later than 65 years old, the usual threshold age for being defined as “elderly”. Few molecular studies have been published on this topic, reporting compositional differences between groups of young adults (20–50 years old) and elderly with a wide interval of age, ranging from 60 to 95 years old [14], [16], [17]. Thanks to a narrower range of age for the recruited elderly (63–78 years old), and the introduction of an additional time-point by analyzing the centenarians, we now provide a higher resolution on the effects of ageing on the gut microbiota. Our comprehensive approach indicates that the threshold for an “aged” microbiota should be moved to the age of 75–80 years.

In the recent description of the HITChip technology an age-related increase in the number of Bacilli, and a decrease in bacterial groups belonging to Bacteroidetes were reported in elderly adults with an average age of 71 years [20]. Interestingly, the present, more extensive HITChip study with a considerably larger sample set, revealed only centenarians to contain significantly higher proportions of Bacilli, while the Bacteroidetes population remained unchanged among the age groups. A possible explanation of this discrepancy may reside in the demographic and geographic differences between the study population, also defined as country specificity, which has been reported to strongly affect the age-related changes in the gut microbiota composition [16].

Our experimental data show that, after 100 years of symbiosis with its host, the gut ecosystem is characterized by a lower relative contribution of *Clostridium* cluster XIVa than in younger people. This decrease has already been described as an effect of the ageing process [14], [32], [33]. Furthermore, our results indicate that the proportion of *Clostridium* cluster IV in the total microbiota is comparable in all the subjects, but the proportions of different phylotypes within the cluster differ significantly between the age groups, suggesting a rearrangement in the composition of this *Clostridium* cluster in centenarians. In particular, we observed a rearrangement in the population of butyrate-producing bacteria in centenarians. Butyrate is a short chain fatty acids mainly produced in the gut by Firmicutes of the *Clostridium* clusters IV and XIVa [34]–[36], which is receiving a growing interest in the gut ecology, as it represents a major energy source for the enterocytes and has been implicated in the protection against inflammatory bowel diseases [37]. Several butyrate producers were found in lower amounts in centenarians than in the other age groups. These included *Ruminococcus obeum et rel., Roseburia intestinalis et rel., E. ventriosum et rel., E. rectale et rel., E. hallii et rel*. (all belonging *Clostridium* cluster XIVa), and *Papillibacter cinnamovorans et rel.*, and *F. prausnitzii et rel.* (*Clostridium* cluster IV). Conversely, the butyrate producers *Anaerotruncus colihominis et rel.* (*Clostridium* cluster IV), and *Eubacterium limosum et rel. (Clostridium* cluster XV) increased in centenarians. The increase of *E. limosum* is high (approximately 15-fold), and could point to a group of bacteria characteristic of the long life. Also the decrease of *F. prausnitzii* in centenarians is of interest as this species is known to affect the gut inflammation processes, exerting a butyrate-independent anti-inflammatory effect [38], [39].

Another feature of the centenarians' gut ecosystem is the increase in facultative anaerobes, such as bacteria belonging to the groups *Fusobacterium*, *Bacillus*, *Staphylococcus*, *Corynebacterium*, *Micrococcaceae*, and many members of the phylum Proteobacteria. Such opportunistic species, especially *Enterobacteriaceae*, thrive in an inflamed environment [40], [41], and are known to increase in elderly [10].

We detected a significant decrease of bifidobacteria in centenarians when compared to young adults, which is another well reported effect of age [15], [16]. Interestingly, we found increased levels of the mucin degrading *A. muciniphila* in aged people if compared to the young adults. This contrasts with the earlier results obtained by Collado *et al* [42], who demonstrated with FISH and PCR that *A. muciniphila* counts slightly decline with the age. There is evidence for the presence of several *A. muciniphila*-like phylotypes in the gut and it is possible that these are detected by the HITChip hybridization but not with specific qPCR primers [43]. Alternatively, this discrepancy may be explained by the country-specificity effects, as well as differences in the diet of the aged people, that may favour populations of bacteria able to degrade mucins.

It is evident that the fecal microbiota of centenarians shows all the features of a partially compromised ecosystem, whose balance with the immune system is likely to be upset. The immune system of these very old people deteriorates as a result of the immunosenescence. The immuno-phenotyping allowed us to confirm that the major, expected age-related changes of the T cells compartment are present in the enrolled subjects [44]–[48]. This suggests that the population here considered represents a suitable sample to investigate the possible relationship between gut microbiota composition and immune/inflammatory status. In fact, in our study population, the proportion of centenarians showing a high inflammation score was significantly higher than in the other age groups, confirming the inflammageing hypothesis [6], [7], [49]. The parallel analysis of the gut microbiota composition and the inflammatory parameters shows that the increase in pro-inflammatory cytokines in the peripheral blood correlates with changes in the gut microbiota profile of centenarians. In particular, the increase of IL-6 and IL-8, which is expected for these exceptionally old subjects in spite of their reasonably good health status [7], [50], [51], was linked with an enrichment in Proteobacteria and a decrease in the amount of some butyrate producing bacteria, such as *E. rectale*, *E*. *hallii*, and *E. ventriosum*. This observation supports the hypothesis that the age-related differences in the gut microbiota composition, particularly evident between centenarians and the other age groups (i.e. proliferation of opportunistic Proteobacteria at the cost of symbionts *Firmicutes* and *Bacteroidetes*), may either contribute to inflammageing or be affected by the systemic inflammatory status. This is supported by recent findings demonstrating that inflammation shifts the balance between symbionts and pathobionts, to the side of the latter [29]. However, despite their inflammatory status, centenarians reached the extreme limit of human lifespan, and escaped or delayed the major age-related diseases which share an inflammatory pathogenesis. This suggests that the inflammatory status may have been counterbalanced by other physiological events, to help the survival of these exceptional individuals. As for the gut microbiota, we demonstrated that there is a remodelling in the composition of the Firmicutes population in centenarians. Among the decreasing Firmicutes, we found many species known to have anti-inflammatory properties, but others with unknown anti-inflammatory properties may be among the increasing ones. For instance, the remarkable increase of *E. limosum* in centenarians could be of particular interest, opening the way to the fascinating hypothesis that *E. limosum* may positively contribute to the health status of an aged and inflamed intestine due to its anti-inflammatory properties [52], and its ability to convert dietary isoflavonoids into other phytoestrogens [53], [54]. Phytoestrogens are being intensively studied because of their potential protective role against the development of several cancer, and coronary heart diseases [55].

In conclusion, the differences in the gut microbiota that we detected between centenarians and the other adults could support the possibility that, to attain longevity, a complex and pervasive remodelling, including that of gut microbiota, should occur, favouring the balance between inflammatory and anti-inflammatory processes. With the growing life expectancy of the human beings, it is estimated that the elderly population (over-60 years old) will reach 2 billion by 2050 [56]. Consequently, the quality of life of this increasing share of the human population is going to become an imperative concern, also in relation to the health-care cost. Maintenance of the homeostasis in the gut ecosystem could be essential for the gut health during the ageing process, and, in this perspective, dietary manipulation of the gut microbiota of the elderly may represent a mean for preserving a healthy gastrointestinal microbial community.

## Materials and Methods

### Subjects and study groups

Eighty-four subjects belonging to different age groups were enrolled for this study in Emilia Romagna, which includes Bologna city and surrounding area, Italy. Group C was composed of 21 centenarians (20 women, 1 men) aged 99 to 104 years (average 100.5). Group E was composed of 22 elderly (11 women, 11 men) aged 63 to 76 years (average 72.7) genetically unrelated to the centenarians in group C, and they were offspring of parents who did not reach longevity (average parents death age, 59.3). Group Y was composed of 20 young adults (9 women, 11 men) aged 25 to 40 years (average 31). Group F, offspring of the centenarians belonging to group C, composed of 21 elderly people (10 women, 11 men) aged 59 to 78 (average 67.5), was also included in the analysis. The study protocol was approved by the Ethical Committee of Sant'Orsola-Malpighi University Hospital (Bologna, Italy). After obtaining written informed consent, a standard questionnaire to collect information regarding the health status, drugs use, clinical anamnesis, and life style was administrated. Subjects of groups E, F, and Y were non-institutionalized and living in their own household. They all showed good physical and cognitive health conditions, as evaluated by tests to assess cognitive ability (Mini Mental State Examination, MMSE), self sufficiency and autonomy (ADL, IADL), physical ability (Handgrip Test and Chair Stand Test), which are used by geriatricians to define the physical and cognitive health status (these data are summarized in Supporting Table S1). The physical and cognitive health status of centenarians in group C mirrors that of the majority of Italian centenarians, as previously characterized by Franceschi *et al* [22]. Subjects affected by malignant neoplasia and/or in therapy with immunosuppressive drugs like cyclosporin, methotrexate, glucorticoids, anticoagulant drugs, and who recently (at one month) used antibiotics were excluded from the study. Subjects in group Y were drug-free, whereas older subjects (groups C, E and F) were assuming a variety of medication as it is common for elderly (Supporting Table S1). Arterial blood pressure measurement, and Body Mass Index (BMI) calculation were also performed. Peripheral blood and feces were collected from each subject. Blood samples were not collected from 3 centenarians and 1 subject in group F because of refusal or impossibility of the subjects. Principal haemato-biochemical parameters evaluation and immunophenotypical analyses were performed on freshly collected blood samples. The resulting plasma samples were stored at −80°C for less than 3 months, and used for the cytokines pattern evaluation. Feces were stored at −80°C and analyses were performed within 3 months.

### DNA extraction from feces

Total microbial DNA was extracted using QIAamp DNA Stool Min Kit (Qiagen, Duesseldorf, Germany) with a modified protocol. 250 mg of feces were suspended in 1 ml of lysis buffer (500 mM NaCl, 50 mM Tris-HCl pH 8, 50 mM EDTA, 4% SDS). Four 3 mm glass beads and 0.5 g of 0.1 mm zirconia beads (BioSpec Products, Bartlesville, OK) were added, and samples were treated in FastPrep (MP Biomedicals, Irvine, CA) at 5.5 ms for 3 min. Samples were heated at 95°C for 15 min, then centrifuged for 5 min at full speed to pellet stool particles. 260 µl of 10 M ammonium acetate were added to the supernatant, followed by incubation in ice for 5 min and centrifugation at full speed for 10 min. One volume of isopropanol was added to each sample and incubated in ice for 30 min. Precipitated nucleic acids were collected by centrifugation for 15 min at full speed and washed with ethanol 70%. Pellets were suspended in 100 µl of TE buffer and treated with 2 µl of DNase-free RNase (10 mg/ml) at 37°C for 15 min. Protein removal by Proteinase K treatment and DNA purification with QIAamp Mini Spin columns were performed following the kit protocol. Final DNA concentration was determined by using NanoDrop ND-1000 (NanoDrop® Technologies, Wilmington, DE).

### 16S rRNA gene amplification

The total bacterial 16S rRNA genes were amplified from fecal DNA using FastStart Taq DNA polymerase, dNTPack PCR Amplification kit (Roche, Mannheim, Germany). *T7prom*-Bact-27-F and Uni-1492-R primer set was used for amplification [20], [57]. PCR reactions were carried out as previously described [20], using 20 ng of DNA as template. Per DNA sample 2 reactions were performed, pooled, and purified by using High Pure PCR Clean up Micro kit (Roche), following the manufacturer instructions.

### RNA preparation

*In vitro* transcription of T7-modified 16S rRNA amplicons was carried out as previously described [20]. Ammino-allyl modified rUTP (Ambion, Austin, TX) was incorporated during the transcription to allow the following CyDye incorporation. RNA was purified using the RNeasy Mini-Elute Kit (Qiagen) according to the manufacturer instructions. Ammino-allyl-modified nucleotides were coupled with CyDye using Post Labelling Reactive Dye (Amersham Bioscience, Piscataway, NJ).

### HITChip analysis

HITChip slides were custom synthesized by Agilent Technologies (Wilmington, DE), having a 8×15K format with 8 arrays per slides. Two different RNA samples, labelled with Cy3 and Cy5 respectively, were hybridized on each array. At least 2 hybridizations on different arrays were carried out for each sample. Fragmentation of the labelled RNA and hybridization were carried out as previously described [20]. Microarrays were scanned by using an Agilent Microarray Scanner at 2 UV lamp intensities (40 and 10% of maximum PMT voltage for red, 60 and 20% of maximum PMT voltage for green). Intensity values for each spot were quantified by using Agilent Feature Extraction software, version 9.5.

Data storage, spatial and quantile normalization, and probe profile extraction were performed using a custom-designed MySQL-based relation database (www.mysql.com) and the R statistical software (www.r-project.org) [20]. The reproducibility of the experiments was assessed by calculating Pearson's correlation of the natural logarithm of spatially normalized signals. Hybridizations which resulted in a Pearson's correlation coefficient <0.98 were repeated. For each probe, hybridization signal was calculated as the mean value of the normalized fluorescence intensities obtained in the 2 replicates. Hierarchical clustering of HITChip profiles was carried out using Pearson's correlation to calculate the distance among samples and Ward's minimum variance as agglomeration method.

For quantification purpose, the analysis was focused on the 129 “level 2” (termed here “genus-like”) phylogenetic groups, corresponding to subsets of phylotypes with 90% or more 16S rRNA sequence similarity [20]. The average signal intensity of all probes which are specific for one phylogenetic group was used as quantitative measure of that group in the sample. Cut-off values for positive responding probes were calculated as described by Rajilic-Stojanovic *et al* [20].

Statistical analysis was performed on log-transformed data using the R software and the Canoco package for Windows [58]. To evaluate the significance of the difference between datasets, P value was calculated by Student t-test for normally distributed data, or Wilcoxon rank sum test for not-normally distributed data. Simpson's reciprocal index of diversity [59], was used to express the diversity of microbial communities, calculated using the equation λ = 1/Σ Pi^2^, where Pi is the proportion of the i^th^ taxon. The proportion of each taxon was calculated as the proportion of each probe signal compared to the total signal. A higher value of Simpson index corresponds to a more diverse community.

### Quantitative PCR

Quantitative PCR (qPCR) analysis was carried out in a Applied Biosystems 7300 Fast Real-Time PCR System (software version 1.4.0), using SYBR Green chemistry (Power SYBR Green PCR Master Mix, Applied Biosystems, Foster City, CA) and 0.2 µM primer concentration. The thermocycling conditions and universal primers for bacteria and specific primers for *Clostridium leptum* group, *Bifidobacterium* genus, *Akkermansia muciniphila*, *Faecalibacterium prausnitzii* and for the genus *Methanobrevibacter*, representing a dominant archaeon in the human gut bacteria have been described previously [42], [60]–[65]. The qPCR reaction volume was 25 µl, employing 100 ng of DNA sample as template. Samples were assayed in duplicate in 2 independent runs. Melting curve analysis was used to confirm specificity of amplification. The amount of 16S rRNA gene copies of specific bacterial groups in the fecal samples was determined by comparing the Ct (threshold cycle) values of samples to those of the standard curves. The standards were prepared by amplifying the 16S rRNA gene from a representative species from each bacterial group, purifying the amplicons with the QIAquick PCR purification kit (Qiagen), and measuring the concentration spectrophotometrically. Appropriate dilutions with a known number 16S rRNA gene copies were used as standards. Statistical analysis of qPCR data was carried out with log-transformed data. Non-detected values were imputed with the half of the theoretical detection limit. Nonparametric tests were used since data were not normally distributed. Kruskall-Wallis test was used to determine the statistical differences among the age groups. Mann-Whitney U-test was used for pair-wise comparisons.

### Immunophenotype characterization

Identification of the major lymphocyte subsets (B lymphocytes, T lymphocytes, virgin T lymphocytes, memory T lymphocytes and NK cells) was performed using the following combination of monoclonal antibodies: CD5/CD19 to identify B and autoreactive B lymphocytes, CD3/CD4/CD8 to identify T lymphocytes subsets, CD28/CD95/CD4 and CD28/CD95/CD8 to identify effector T helper and cytotoxic lymphocytes, CD45RA/CD4/CCR7 and CD45RA/CD4/CCR7 to identify naïve, central memory, effector memory and terminal effector T helper and cytotoxic lymphocytes and CD4/CD25/CD8 to identify activated T lymphocytes. Phenotypical analysis of peripheral blood lymphocytes on whole blood samples was performed as previously described [44], [47]. A cytometric approach with fluorochrome-labelled monoclonal antibodies (mAbs, BD Biosciences Pharmingen, San Diego, CA) directly conjugated to fluorescent molecules (fluorescein isothiocyanate, FITC; phycoerythrin, PE; phycoerythrin-cy-crhome, PE-Cy7) staining was used. Phenotypic analyses of cytotoxic and helper subsets were performed placing an electronic gate on CD8^+^ bright and CD4^+^ bright cells and evaluating the expression of CD45RA versus CCR7. An example of the gating strategy is reported in Supporting Figure S2. Similarly, the expression of CD25 was analyzed on CD8^+^ bright and CD4^+^ bright gate. A minimum of 30,000 cells per sample was acquired using a FACScalibur (BD Biosciences) flow cytometer and data were analyzed with FlowJo (Tree Star Inc., Ashland, OR) software.

### Evaluation of plasma cytokines levels

The concentrations of IL-1α, IL-1β, IL-2, IL-6, IL-8, IL-10, IL-12p70, IFN-γ, and TNF-α trimer in plasma samples of all enrolled subjects were measured in duplicate by multiplex sandwich ELISA technology (Human Cytokine Array 1, SearchLight, Aushon Biosystems, Billerica, MA) according to the manufacturer's instructions. Plasma TGF-β1 concentration was determined in duplicate by ELISA using a commercial kit (DRG Instruments GmbH, Marburg, Germany) according to the manufacturer's instructions.

### Inflammation score

To evaluate the inflammatory status of each subject, an Inflammation Score (IS) was calculated as inspired by previous studies [66], [67]. The score was composed of 8 parameters: white blood cell count, C reactive protein, number of central memory helper and cytotoxic T lymphocytes (CD45RA^−^CCR7^+^), number of effector helper and cytotoxic T lymphocytes (CD28^−^), IL-6 and IL-8 plasma levels. IS, ranging from 0 to 8, was calculated by adding 1 unit for each value greater than the median of the study samples for each inflammatory parameter. Subjects with IS value between 5 and 8 were considered highly inflamed.

## Supporting Information

Figure S1 Score plot of the PCA, centered by species and grouped by samples, of the microbiota composition of centenarians (C, green circles), elderly (E, blue squares), and offspring of the centenarians (F, pink boxes). Explanatory variables are indicated by red triangles. First and second ordination axes are plotted, explaining together the 31% of the variability in the considered dataset. Log transformed data were used for the analysis.
(0.35 MB TIF)

Figure S2 Cytofluorimetric strategy used to identify naïve and memory CD4+ T cells (an example). Lymphocytes were gated according to their physical parameters (FSC and SSC) and then selected for CD4+bright cells, which were subdivided using CD45RA and CCR7. Thus, the following subsets were identified: naïve CD4+ bright T lymphocytes (CD45RA+CCR7+), central memory CD4+ bright T lymphocytes (CD45RA-CCR7+), effector memory CD4+ bright T lymphocytes (CD45RA-CCR7-), terminal effector memory CD4+ bright T lymphocytes (CD45RA+CCR7-).
(0.14 MB PDF)

Table S1 Health status and medication of centenarians, offspring of centenarians, and unrelated elderly subjects included in the study.
(0.05 MB PDF)
