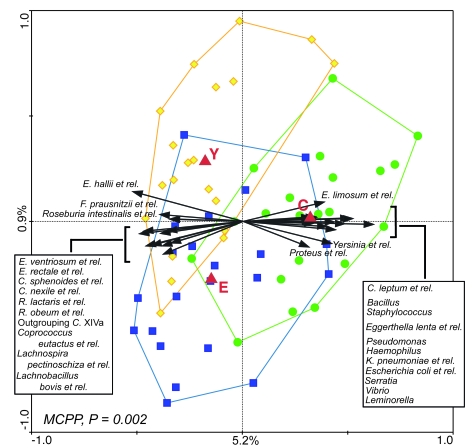# Correction: Through Ageing, and Beyond: Gut Microbiota and Inflammatory Status in Seniors and Centenarians

**DOI:** 10.1371/annotation/df45912f-d15c-44ab-8312-e7ec0607604d

**Published:** 2010-06-08

**Authors:** Elena Biagi, Lotta Nylund, Marco Candela, Rita Ostan, Laura Bucci, Elisa Pini, Janne Nikkïla, Daniela Monti, Reetta Satokari, Claudio Franceschi, Patrizia Brigidi, Willem De Vos

Figure 2 contains a typographic error. One of the centroid labels should be "E" instead of "K". Please view the correct figure here: 

**Figure:**