# Supplementary figures and images for: Through Ageing, and Beyond: Gut Microbiota and Inflammatory Status in Seniors and Centenarians
Source: PLoS One. 2010 May 17;5(5):e10667. doi: 10.1371/journal.pone.0010667 (PMC2871786; doi:10.1371/journal.pone.0010667)

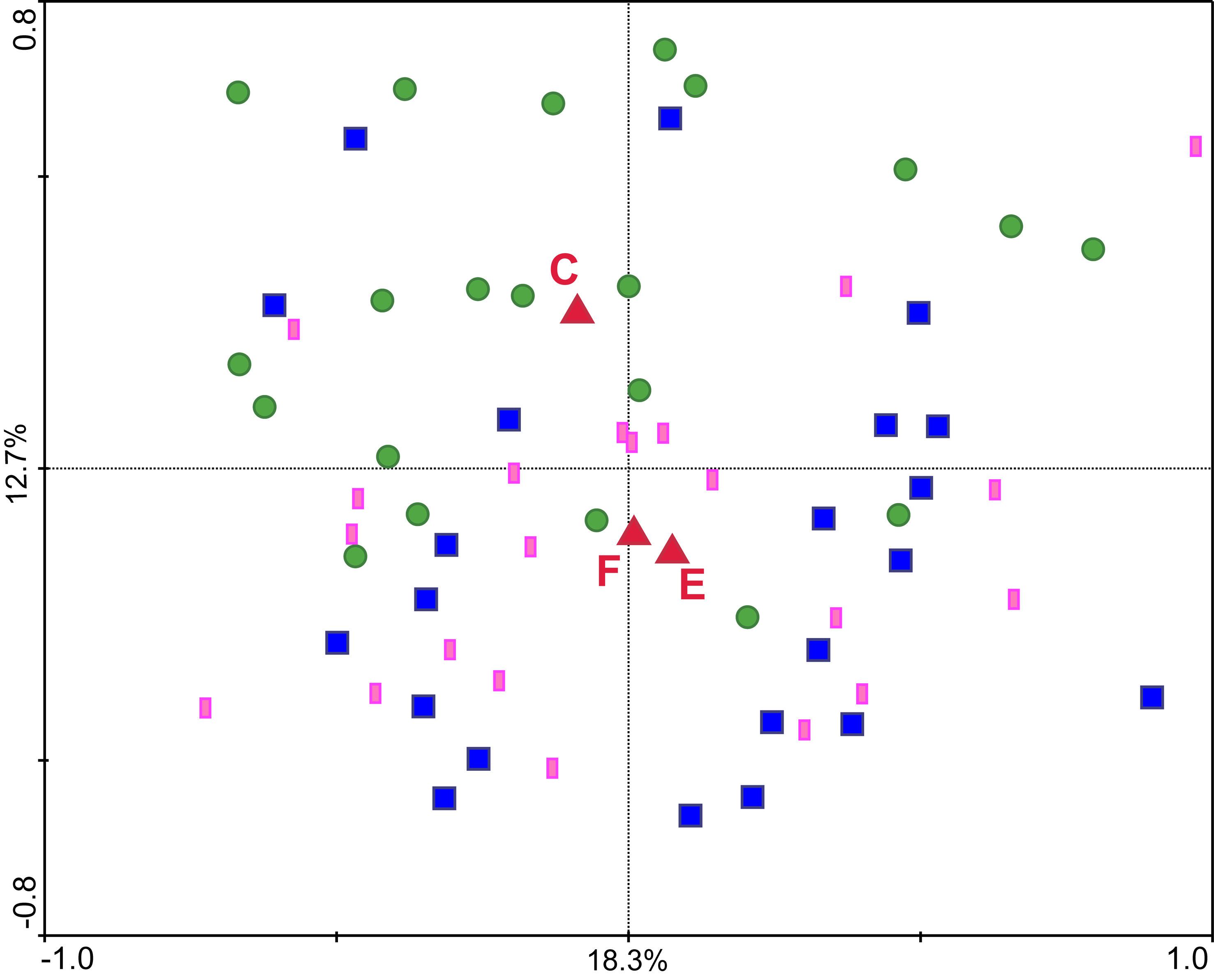

Supplement: Figure S1 — Score plot of the PCA, centered by species and grouped by samples, of the microbiota composition of centenarians (C, green circles), elderly (E, blue squares), and offspring of the centenarians (F, pink boxes). Explanatory variables are indicated by red triangles. First and second ordination axes are plotted, explaining together the 31% of the variability in the considered dataset. Log transformed data were used for the analysis. (0.35 MB TIF) [file pone.0010667.s001.tif]
