# Supplementary material for: Through Ageing, and Beyond: Gut Microbiota and Inflammatory Status in Seniors and Centenarians
Source: PLoS One. 2010 May 17;5(5):e10667. doi: 10.1371/journal.pone.0010667 (PMC2871786; doi:10.1371/journal.pone.0010667)
Supplement: Figure S2 — Cytofluorimetric strategy used to identify naïve and memory CD4+ T cells (an example). Lymphocytes were gated according to their physical parameters (FSC and SSC) and then selected for CD4+bright cells, which were subdivided using CD45RA and CCR7. Thus, the following subsets were identified: naïve CD4+ bright T lymphocytes (CD45RA+CCR7+), central memory CD4+ bright T lymphocytes (CD45RA-CCR7+), effector memory CD4+ bright T lymphocytes (CD45RA-CCR7-), terminal effector memory CD4+ bright T lymphocytes (CD45RA+CCR7-). (0.14 MB PDF) [file pone.0010667.s002.pdf]

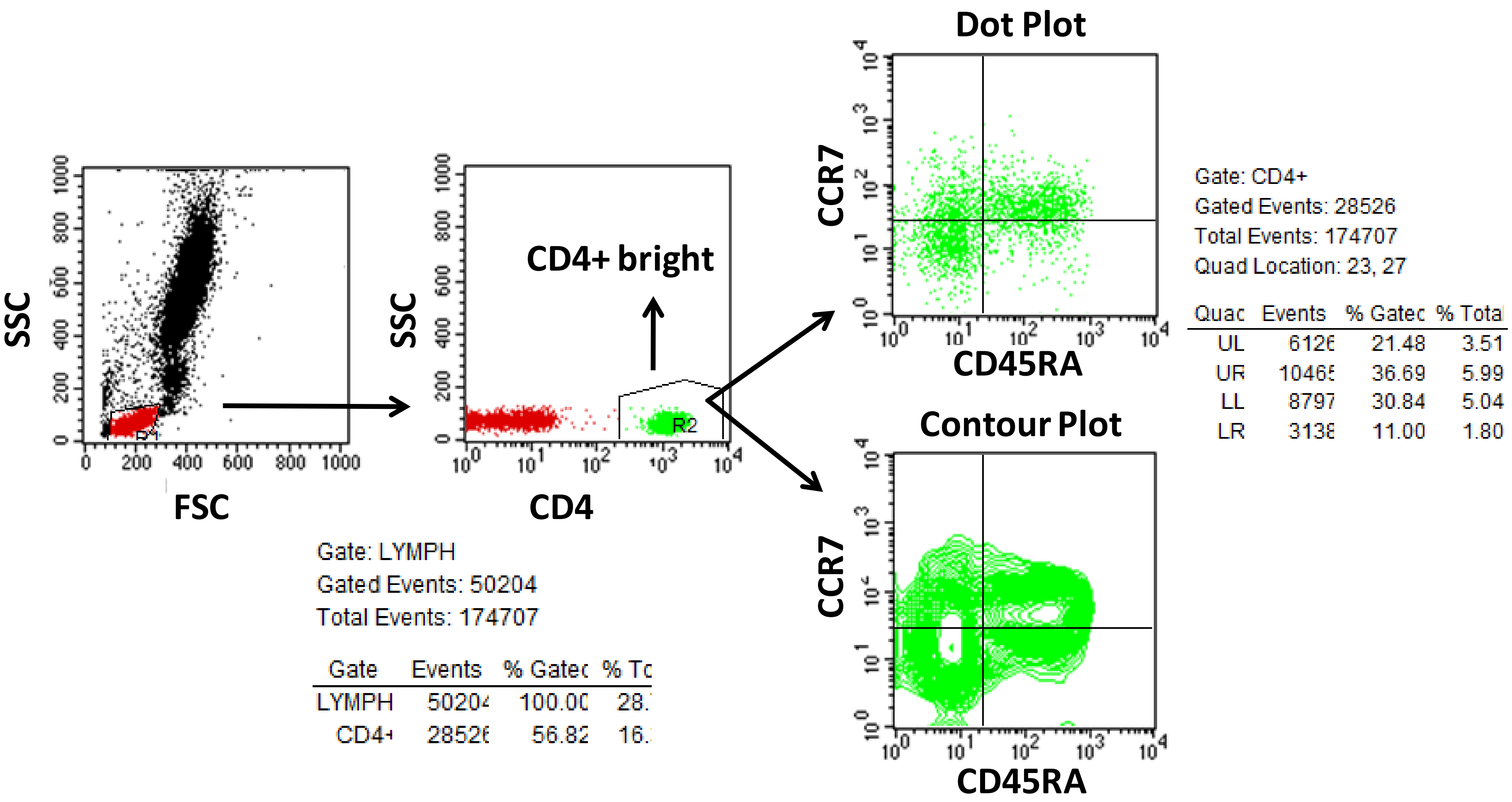
SSC
FSC
R1
SSC
CD4
CD4+ bright
R2
Gate: LYMPH
Gated Events: 50204
Total Events: 174707
Gate Events % Gatec % Tc
LYMPH 5020 100.0C 28.
CD4 2852 56.82 16.
Dot Plot
CCR7
CD45RA
Contour Plot
CCR7
CD45RA
Gate: CD4+
Gated Events: 28526
Total Events: 174707
Quad Location: 23, 27
Quac Events % Gatec % Total
UL 612 21.48 3.51
UR 1046 36.69 5.99
LL 8797 30.84 5.04
LR 313 11.00 1.80
