# Supplementary material for: Through Ageing, and Beyond: Gut Microbiota and Inflammatory Status in Seniors and Centenarians
Source: PLoS One. 2010 May 17;5(5):e10667. doi: 10.1371/journal.pone.0010667 (PMC2871786; doi:10.1371/journal.pone.0010667)
Supplement: Table S1 — Health status and medication of centenarians, offspring of centenarians, and unrelated elderly subjects included in the study. (0.05 MB PDF) [file pone.0010667.s003.pdf]

Supporting Table S1. Health status and medication of centenarians, offspring of centenarians, and unrelated elderly subjects included in the study[a].

HEALTH STATUS	**Centenarians** (C, n=21)	**Elderly** (E, n=22)	**Offspring** (F, n=21)
**Mild Cognitive impairment (18 ≤ SMMSE[b] ≤ 23)**	3	0	0
**Severe Cognitive impairment (SMMSE[b] ≤ 17)**	8	0	0
**SMMSE[a] not assessed for sensory impairment[c]**	4	0	0
**Moderately disabled (ADL[d]: 1-2 disabilities)**	4	0	0
**Severely disabled (ADL[d]: 3-5 disabilities)**	5	0	0
**MEDICATION**			
**Antinflammatory drugs[e]**	3	3	4
**Cardiovascular therapy**	15	6	4
**Antihypertensive therapy**	10	12	7
**Antidiabetic Therapy**	1	3	0
**Drugs affecting lipoprotein metabolism[f]**	0	11	4
**Micronutrients o vitamins**	4	1	1
**Laxatives**	1	0	0
**Liver-biliary therapy**	0	1	1
**Gastro-intestinal disorders drugs**	5	1	0
**Acid secretion drugs**	8	4	2
**Gout medication**	1	1	0
**Osteoporosis drugs**	1	5	2
**Thyroid therapy**	0	3	0
**Miscellaneous central nervous system agents**	6	8	5
**Respiratory agents**	1	5	0
**Vasoprotective agents**	1	1	0

[a]Young subjects were all in good physical and free of medication.
[b]Standardized Mini Mental State Examination (SMMSE) (Folstein et al, J Psychiatr Res, 1975). The score used in the analysis was corrected by age and years of educations (Magni et al, Int Psychogeriatr, 1996).
[c]These subjects were apparently in good cognitive condition.
[d]Index of Independence in Activities of Daily Living (adapted from Katz et al, Gerontologist, 1970).
[e]Nonsteroidal Antiinflammatory Drugs (NSAIDs) and Corticosteroids.
[f]Statins and fibric acid.
